# Supplementary material for: Effects of Methyl Sulfonyl Methane and Selenium Yeast on Fatty Liver Syndrome in Laying Hens and Their Biological Mechanisms
Source: Animals (Basel). 2023 Jul 30;13(15):2466. doi: 10.3390/ani13152466 (PMC10416963; doi:10.3390/ani13152466)
Supplement: Supplementary file 1 [file animals-13-02466-s001.zip › supplementary Table S1.pdf]

Supplementary Table S1: Relevant information of antioxidant kits.

Antioxidant kits	Cargo number	Purchasing company
MDA	A003-1-2	Nanjing Jiancheng Biotechnology Co., Ltd.
CAT	A007-2-1	Nanjing Jiancheng Biotechnology Co., Ltd.
T-SOD	A001-1-2	Nanjing Jiancheng Biotechnology Co., Ltd.
GPX	BC1195	Solarbio Biotechnology Co., Ltd.
